# Professional Brotherhood

**Published:** 1877-04-20

**Authors:** 


### PROFESSIONAL BROTHERHOOD.

It is a matter to be regretted that there does not exist among medical men that degree of social feeling and brotherhood that may be seen amongst members of the legal profession, or other callings. This should not be so. Medical men in every community should foster and encourage a kindly and social feeling for each other. It is a mistake to suppose that one can the better promote his business interest by envy and opposition to business competitors. The opposite is true and in the end proves to be the best policy. Medical societies, by throwing physicians often together, are calculated to drive off jealousies and promote social feeling. We, therefore, recommend our medical brethren everywhere, whether in city, village, or country to organize medical societies; cultivate a feeling of professional regard and brotherhood; talk with each other; talk for each other, and work for each other, and you will find it best for yourselves, best for your patrons, and best for the profession at large.

